# Effectiveness of Individually Trained Oral Prophylaxis (iTOP) Education on Long-Term Oral Health in Medical and Dental Students: A Two-Year Prospective Cohort Study

**DOI:** 10.3390/dj13090404

**Published:** 2025-09-03

**Authors:** Zvonimir Lukac, Brigita Maric, Josip Kapetanovic, Mislav Mandic, Ivona Musa Leko, Andrija Petar Bosnjak

**Affiliations:** 1 Department of Periodontology, School of Medicine, University of Mostar, 88000 Mostar, Bosnia and Herzegovina; josip.kapetanovic@mef.sum.ba (J.K.); ivona.musa-leko@mef.sum.ba (I.M.L.); andrija.bosnjak@mef.sum.ba (A.P.B.); 2 Department of Prosthodontics, School of Medicine, University of Mostar, 88000 Mostar, Bosnia and Herzegovina; brigita.maric@mef.sum.ba (B.M.); mislav.mandic@mef.sum.ba (M.M.); 3 Polyclinic Musa, 88000 Mostar, Bosnia and Herzegovina; 4 Dental Polyclinic Arena, Adria Dental Group, 10000 Zagreb, Croatia

**Keywords:** oral health, oral hygiene, iTOP, oral health education, preventive dentistry, medical students, dental students

## Abstract

**Background/Objectives**: Preventive oral health education plays a key role in preparing future healthcare professionals to promote and maintain good oral hygiene. Individually Trained Oral Prophylaxis (iTOP) is a structured, personalized educational program that emphasizes correct brushing techniques and interdental cleaning. This study aimed to evaluate the long-term effectiveness of a single-session iTOP intervention on clinical oral health outcomes among medical and dental students. **Methods**: A 2-year prospective cohort study included 82 first- and fourth-year medical and dental students at the University of Mostar, Bosnia and Herzegovina. The researchers randomly assigned participants to an iTOP intervention group or a control group. The primary analysis used multivariable linear mixed-effects models for repeated measures, adjusted for study program, academic year, smoking status, and baseline oral-hygiene habits, with effect sizes reported alongside 95% confidence intervals. Clinical periodontal parameters—plaque index, bleeding on probing, and probing depth—were assessed at baseline, three months, and two years. All participants received professional cleaning and oral hygiene kits. Only the intervention group received personalized iTOP training, consisting of a single session with brief reinforcement at the 3-month follow-up. This study was registered at ClinicalTrials.gov (NCT07085013). **Results**: Seventy-six students completed the follow-up. The iTOP group had significantly lower plaque index and bleeding scores at both follow-up points (*p* < 0.001) compared to the control group. Baseline differences were observed between subgroups (medical vs. dental; younger vs. older students), but these diminished over time. At the 2-year follow-up, only the plaque index remained significantly improved, while other clinical parameters returned to values comparable to baseline. **Conclusions**: The iTOP program resulted in significant short-term improvements in oral health among medical and dental students. For sustained long-term outcomes, iTOP or similar structured oral health education programs should be integrated into medical and dental curricula. Enhancing oral health awareness among healthcare providers may ultimately contribute to improved public oral health outcomes. Given the single-center design and the single-session nature of the intervention, the results should be interpreted with caution.

## 1. Introduction

Oral health is an essential component of general health and quality of life, affecting both physical and psychosocial well-being. In recent decades, dentistry has shifted its focus from treatment of oral diseases to prevention. Proper oral hygiene is now recognized as a key preventive measure against common conditions such as dental caries and periodontitis.

Individually Trained Oral Prophylaxis (iTOP) is a personalized approach that promotes oral hygiene through one-on-one instruction and motivation. Although general oral health education shows some efficacy, the long-term benefits of individualized interventions such as iTOP remain insufficiently explored. Some studies suggest that improvements may diminish without continuous reinforcement, while others report sustained positive outcomes after personalized training [1,2,3]. Similar findings have been reported in various populations, including hospitalized elderly patients, suggesting broader applicability [4]. This inconsistency highlights the need for further research, especially among medical and dental students, who often differ in baseline knowledge and oral health behaviors [5]. A study conducted among dental students at the University of Zagreb showed that although oral health knowledge improves with academic progression, oral hygiene behaviors may not follow the same trend, highlighting the importance of sustained motivation and self-care training throughout the curriculum [6]. In addition, a study among adolescents in Bosnia and Herzegovina found significant sex-based differences in oral health behaviors and perceptions, yet found no correlation with socioeconomic status, underscoring the complexity of behavioral factors that influence oral health [7].

Oral health has also been linked to systemic conditions such as cardiovascular and metabolic diseases, further emphasizing the importance of prevention [8].

Despite advances in dental care, the global burden of caries and periodontitis remains high and highlights the need for effective, sustainable educational strategies [9,10]. Medical and dental students are an important target group for oral health promotion, as they represent future healthcare professionals. However, studies have shown that even medical and dental students often exhibit suboptimal hygiene habits and inconsistent oral health behaviors, indicating a persistent gap between knowledge and practice [5,6,11,12,13,14]. This paradox makes them a particularly relevant population for evaluating the effectiveness of structured interventions such as iTOP.

Considerable variability in oral health knowledge, attitudes, and behaviors has been consistently observed among university students. Research from Split, Croatia, conducted among non-healthcare students, highlighted gaps in oral hygiene awareness and practice [11]. A study from Saudi Arabia involving both healthcare and non-healthcare students identified significant disparities in oral health behaviors [12]. The link between oral and mental health has received increasing attention, with cross-sectional studies from Serbia and South Korea reporting associations between stress, self-perceived oral health, and oral symptoms among university student populations [13,14]. These insights advocate for a more holistic and psychologically informed approach to oral health education.

Therefore, this study aimed to evaluate the long-term effectiveness of a single-session iTOP intervention among medical and dental students by assessing clinical periodontal parameters over a 2-year period. Understanding these effects may help guide how structured oral health education is integrated into medical and dental curricula.

Recent systematic reviews in adult populations have shown that educational and behavioral interventions can significantly improve periodontal health. One review focusing on periodontitis patients reported that tailored oral hygiene strategies—including psychological and personalized instruction—enhanced plaque control and gingival outcomes, although heterogeneity prevented a quantitative synthesis [15]. Another systematic review targeting older adults (aged 60+) and their caregivers identified that health education programs led to measurable improvements in oral hygiene and related clinical parameters [16]. These findings underscore the relevance of individualized, single-session instruction and the need to evaluate its long-term clinical impact in adult student cohorts.

## 2. Materials and Methods

### 2.1. Study Design and Participants

This was a prospective cohort study with random allocation of participants to intervention and control groups, constituting a quasi-experimental design. This study followed STROBE guidelines for observational studies and incorporated relevant CONSORT elements in reporting randomization. It was conducted at the University of Mostar School of Medicine (Bosnia and Herzegovina) over two academic years, from the 2021/2022 to the 2023/2024 academic year. This study was registered at ClinicalTrials.gov (NCT07085013). The sample included all eligible first- and fourth-year medical and dental students enrolled in 2021/2022 (*n* = 82), who were followed until their third and sixth years, respectively. A total of 76 participants completed all three assessments (baseline, 3 months, and 2 years). Although a formal a priori power analysis was not conducted, we included the entire accessible population. A post hoc power analysis based on the observed effect sizes indicated that the available sample size provided sufficient statistical power (>0.80) to detect moderate-to-large differences in periodontal parameters over time. Previous studies with similar designs and sample sizes also reported adequate sensitivity to detect clinically relevant changes [1,17,18]. Medical and dental students were selected because of their dual role as learners and future healthcare professionals, whose oral hygiene behaviors carry broader public health implications. This makes them a relevant population for evaluating the impact of structured educational interventions.

This study adhered to the Declaration of Helsinki and was approved by the Ethics Committee of the University of Mostar School of Medicine (the Approval Nos. 01-I-1827/21 and 01-I-911/24). All participants provided written informed consent prior to enrollment.

### 2.2. Inclusion and Exclusion Criteria

Inclusion criteria were active enrollment in the first and fourth years (2021/2022) or third and sixth years (2023/2024), as confirmed by Faculty Administration records. All participants also provided written informed consent.

Exclusion criteria were lack of informed consent and the presence of systemic conditions known to affect periodontal health, such as uncontrolled diabetes or immunodeficiencies. Additionally, individuals taking medications known to cause gingival overgrowth, including anticonvulsants, calcium channel blockers, or immunosuppressants, were excluded from this study.

### 2.3. Group Allocation and Interventions

Participants were randomly assigned using a computer-generated sequence (Random.org) to either an intervention group (iTOP training) or a control group. The researchers concealed the allocation sequence from the examiners conducting the clinical assessments to minimize potential selection bias. All clinical examinations were jointly conducted by two examiners: a board-certified specialist in periodontology with more than ten years of clinical experience, and a teaching assistant at the Department of Periodontology, University of Mostar. By working together throughout all assessments, the examiners ensured consistent data collection and diagnostic accuracy. Prior to data collection, both examiners reviewed the study protocol and underwent calibration sessions to align clinical measurement techniques.

### 2.4. Study Procedures

#### Assessments Were Conducted at Three Time Points

Baseline (2021/2022): At the first visit, participants completed a general health and oral health questionnaire and underwent a full periodontal examination. The questionnaire included items on general health, smoking status, and detailed oral hygiene habits such as brushing frequency, use of interdental brushes, and dental floss. Smoking status was also recorded again at the 2-year follow-up; however, in the primary LMM analyses, only the baseline smoking status was used as the covariate. Examiners recorded clinical parameters including plaque levels, bleeding on probing, pocket depth, and average attachment level. They were blinded to participants’ academic program (medical or dental) and intervention allocation (iTOP or control) to reduce potential assessment bias. Although the same examiners assessed participants at all three time points, they were not explicitly informed which visits represented follow-up assessments; however, given the repeated participation of the same students, complete blinding to the time point could not be guaranteed. The clinical team took periodontal measurements at six sites per tooth using the UNC-15 probe (Carl Martin AG, Solingen, Germany) and recorded them using the Bern Periodontal Chart. Clinical attachment level was measured in millimeters relative to the cementoenamel junction (CEJ). Negative values indicated that the gingival margin was located coronally to the CEJ, reflecting absence of clinical attachment loss. Bleeding on probing (BOP) was assessed at six sites per tooth (distobuccal, midbuccal, mesiobuccal, distolingual/palatal, midlingual/palatal, and mesiolingual/palatal) using a UNC-15 probe. A site was scored as positive if bleeding occurred within 30 s of gentle probing. BOP was expressed as the percentage of positive sites out of the total number of sites examined. Plaque levels were disclosed using the PCA 222 plaque indicator (Curaden AG, Kriens, Switzerland). After clinical examination, all participants underwent professional dental cleaning with the SonicFlex scaler and the KaVo ProphyFlex 4 air polisher (KaVo, Biberach, Germany). The study team provided a standardized oral hygiene kit (Curaprox toothbrush and interdental brushes; Curaden AG, Kriens, Switzerland) to all participants. After the baseline examination, participants in the iTOP group attended small-group sessions led by a licensed dental professional certified in the iTOP protocol. The iTOP training included instruction in the Modified Bass toothbrushing technique using a manual toothbrush and the “touch-to-teach” method for optimal brush positioning. Participants practiced plaque removal using a manual toothbrush, single-tuft brush, interdental brushes, and dental floss under direct supervision. Disclosing agents were applied to visualize plaque deposits and guide personalized feedback. The baseline iTOP session lasted approximately 45 min and was conducted in small groups of 10 students. Adherence to oral hygiene recommendations was not systematically monitored during follow-up.

Second Examination (Follow-up at 3 Months): Periodontal parameters were re-evaluated following the same clinical protocol as in the baseline visit. Compliance at this visit was complete (100%). As in the initial examination, all participants received a standardized oral hygiene kit (Curaprox toothbrush and interdental brushes; Curaden AG, Kriens, Switzerland). Those in the iTOP group received a brief (approximately 10 min) motivational reinforcement session.

Third Examination (Long-Term Follow-up after Two Years; 2023/2024): Seventy-six participants (92.7% of the initial sample) returned for the final assessment, during which examiners measured periodontal parameters using the same protocol as at baseline. Professional cleaning was repeated, and all participants received a new standardized oral hygiene kit.

### 2.5. Statistical Analysis

We assessed the distribution of all outcomes—average probing depth (APD, mm), average attachment level (AAL, mm), plaque index (%), and bleeding on probing (BOP, %)—using histograms, Q–Q plots, and the Shapiro–Wilk test, and verified homoscedasticity via residual vs. fitted plots. Because plaque and BOP are bounded percentages, they were analyzed on the logit scale after scaling to proportions with a 0.5 continuity correction, whereas APD and AAL were modeled on the raw scale.

Primary analyses were performed using linear mixed-effects models (random intercept for participant) to account for repeated measures (baseline, 3 months, 2 years). Fixed effects included Group (iTOP vs. control), Time, and their interaction (Group × Time). Models were adjusted for study program, academic year, smoking status, and baseline oral-hygiene habits. For plaque and BOP, model estimates were back-transformed to percentage points for interpretation.

We report adjusted marginal means with 95% confidence intervals (CIs), between-group differences at follow-up, and omnibus Group × Time tests. Sensitivity analyses included unadjusted models and non-parametric comparisons. Model assumptions were checked by residual diagnostics. Estimation used restricted maximum likelihood (REML) with Satterthwaite approximation for degrees of freedom. Missing data were handled under the missing-at-random assumption. All tests were two-sided with α = 0.05. Analyses were conducted in SPSS v26 (MIXED procedure) and cross-checked in R (lme4/emmeans).

Effect sizes (Cohen’s *d* with 95% CI) were calculated for the main between-group differences. A post hoc power analysis, based on observed effect sizes and actual sample sizes, is reported in the Section 2.6.

### 2.6. Post Hoc Power Analysis

No a priori sample size calculation was performed, as this study included the entire eligible student population. Post hoc power analyses were conducted for all primary outcomes (plaque index, bleeding on probing, APD, AAL) at each time point, based on observed effect sizes (Cohen’s *d*) and actual sample sizes. These analyses, presented in Appendix A Table A1, Table A2 and Table A3, suggest that this study had adequate statistical power (≥0.80) to detect moderate-to-large differences in plaque index and bleeding on probing. Given the limitations of post hoc power analysis, these results should be interpreted cautiously.

## 3. Results

### 3.1. Differences Between Medical and Dental Students over Time

Table 1 shows differences in clinical periodontal parameters between dental and medical students at all three time points. At baseline, medical students had significantly higher probing depth and average attachment level values than dental students (*p* < 0.05). No significant differences were observed between groups for the other clinical parameters. At the 2-year follow-up, these differences were no longer present for any parameter.

### 3.2. Differences Between Junior and Senior Students over Time

Table 2 shows differences in clinical parameters between younger (first-/fourth-year) and older (third-/sixth-year) students. At baseline, younger students had significantly higher plaque index values than older students (*p* < 0.05). No significant differences were observed between groups for other periodontal parameters at any time point. Differences between academic levels decreased over time.

### 3.3. Intra-Group Changes over Time in the iTOP and Control Groups

Table 3 shows intra-group comparisons of periodontal parameters for the iTOP and non-iTOP groups at baseline, 3-month, and 2-year follow-ups. At the 3-month follow-up, the iTOP group showed significant reductions in probing depth, average attachment level, plaque index, and bleeding on probing compared with baseline (*p* < 0.001). In the control group, significant reductions were observed in probing depth, attachment level, and plaque index, whereas changes in bleeding on probing were not statistically significant. At the 2-year follow-up, the iTOP group maintained significantly lower plaque scores compared to both prior time points. However, probing depth, attachment level, and bleeding on probing increased, returning to values similar to baseline.

In contrast, the non-iTOP group showed deterioration in periodontal status at the 2-year follow-up, with significantly higher probing depth, attachment level, and bleeding on probing compared with both baseline and 3-month values. Despite this, plaque scores remained significantly lower than at baseline.

**Figure 1:**
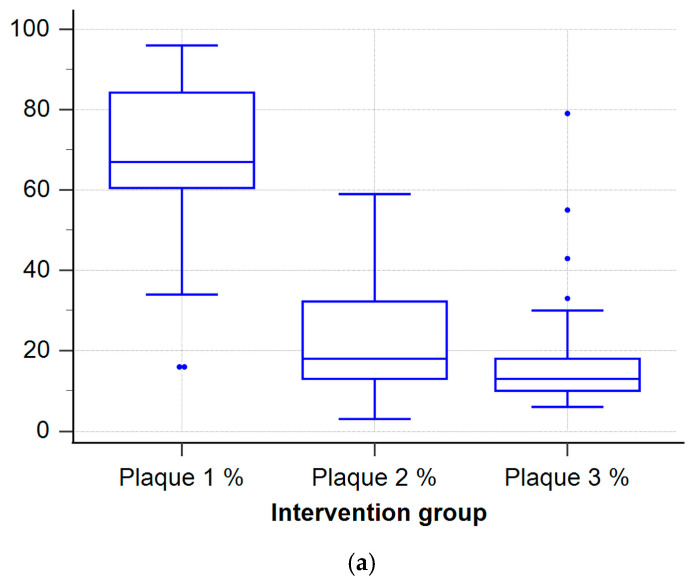

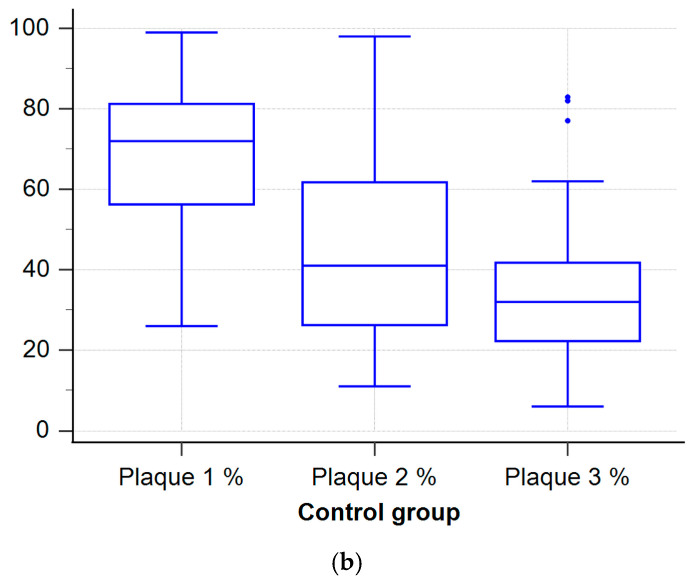
Box-and-whisker plots of plaque index at baseline (Plaque 1), 3-month (Plaque 2) and 2-year (Plaque 3) follow-ups for (**a**) iTOP group and (**b**) non-iTOP group. Boxes show the interquartile range (25th–75th percentile); the horizontal line inside each box is the median; whiskers extend to the most extreme values within 1.5 × IQR. Mean values are shown as blue squares, while outliers are shown as blue dots. Blue color is used only for styling and carries no additional meaning.

Changes in plaque index over three time points were analyzed in both the intervention group (iTOP) and the control group (non-iTOP). Results are shown in box plots (Figure 1a,b) illustrating the distribution, median, interquartile range, and outliers for each group across all time points. In the iTOP group, plaque index significantly decreased from baseline to the 2-year follow-up. In contrast, the non-iTOP group showed a smaller and more variable reduction in plaque index, with values remaining higher than in the iTOP group across all time points.

### 3.4. Inter-Group Comparisons Between iTOP and Control Groups

Table 4 shows between-group comparisons of periodontal parameters at baseline, 3 months, and 2 years. At both the 3-month and 2-year follow-ups, the control group had significantly higher plaque index and bleeding on probing values (*p* < 0.001). No significant between-group differences were found for probing depth or average attachment level at any time point.

### 3.5. Primary Adjusted Analysis

Table 5 shows the results of mixed-effects models adjusted for academic program, academic year, smoking, and baseline oral hygiene habits, where the iTOP group had significantly lower plaque at both 3 months and 2 years compared with controls. At 3 months, the adjusted mean difference was −22.26 percentage points (95% CI: −32.15 to −12.90; *p* < 0.001), and at 2 years it was −15.40 percentage points (95% CI: −24.21 to −7.41; *p* < 0.001). At the 2-year follow-up, the between-group difference in plaque index corresponded to a large effect size (Cohen’s *d* = 1.05, 95% CI: 0.63–1.47). A post hoc power analysis indicated that this study had sufficient statistical power (1 − β = 0.995) to detect this difference.

BOP was also significantly lower in the iTOP group (T2: Δ = −2.70 pp, 95% CI: −4.57 to −1.05; *p* = 0.001; T3: Δ = −5.65 pp, 95% CI: −9.13 to −2.50; *p* < 0.001).

For APD, between-group differences were not statistically significant at either follow-up (T2: Δ = −0.03 mm, 95% CI: −0.10 to 0.05; *p* = 0.456; T3: Δ = −0.02 mm, 95% CI: −0.10 to 0.05; *p* = 0.538). For AAL, no significant difference was observed at 3 months (Δ = 0.03 mm, 95% CI: −0.09 to 0.16; *p* = 0.594), but a small, statistically significant difference was seen at 2 years (Δ = 0.14 mm, 95% CI: 0.00 to 0.27; *p* = 0.043).

Table 6 shows that omnibus Group × Time interaction tests were significant for Plaque (F(2, 240) = 22.41, *p* < 0.001) and BOP (F(2, 240) = 7.88, *p* < 0.001), significant for AAL (F(2, 240) = 3.52, *p* = 0.031) — although the between-group differences were very small (≈0.14 mm at 2 years) and thus of limited clinical relevance—and not significant for APD (F(2, 240) = 0.78, *p* = 0.460).

### 3.6. Trends in Smoking Behavior

**Figure 2:**
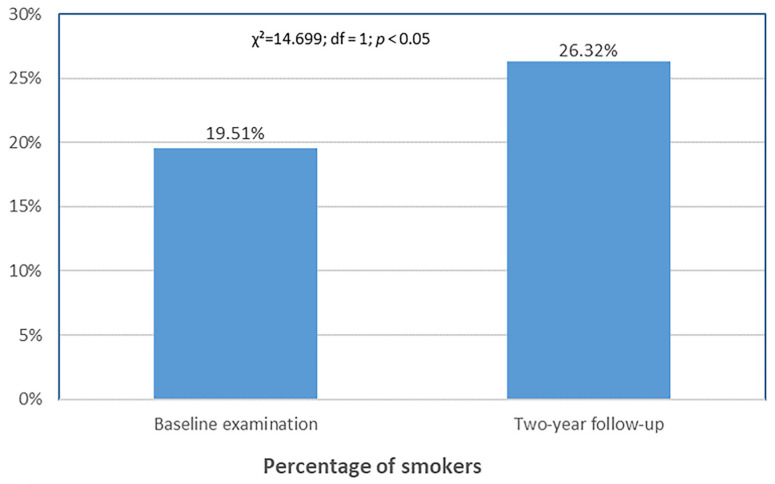
Change in self-reported smoking prevalence over two years.

Figure 2 shows a significant increase in self-reported smoking at the 2-year follow-up compared with baseline (*p* < 0.05).

### 3.7. Self-Reported Oral Hygiene Behaviors

Oral hygiene behaviors were assessed using a self-reported questionnaire administered only at baseline. Participants provided information on daily toothbrushing frequency, and use of dental floss and interdental brushes. These baseline data offer a descriptive overview of the participants’ hygiene habits at the start of this study and provide context for interpreting clinical outcomes.

**Figure 3:**
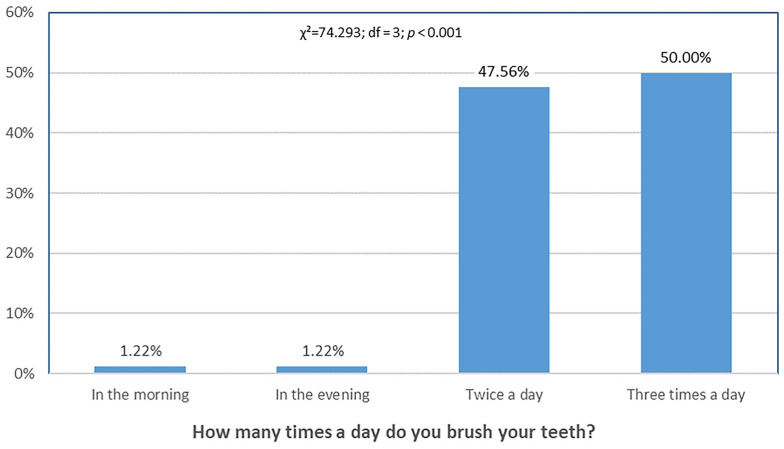
Differences in toothbrushing frequency. Distribution of responses differed significantly across categories (χ^2^ = 74.293, df = 3, *p* < 0.001).

Figure 3 shows that the proportion of participants who brushed their teeth only once per day (morning or evening) was the lowest, with a statistically significant difference compared with other frequencies.

**Figure 4:**
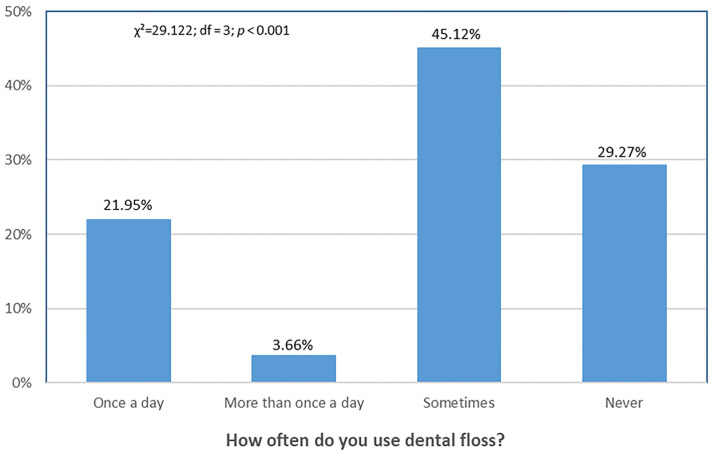
Differences in frequency of dental floss use. Distribution of responses differed significantly across categories (χ^2^ = 29.122, df = 3, *p* < 0.001).

Figure 4 shows that the proportion of participants who used dental floss multiple times per day was the lowest, whereas the highest proportion used dental floss occasionally.

**Figure 5:**
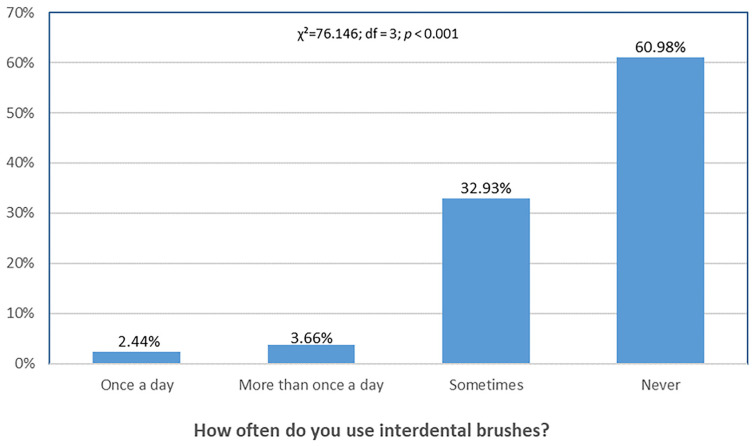
Differences in frequency of interdental brush use. Distribution of responses differed significantly across categories (χ^2^ = 76.146, df = 3, *p* < 0.001).

Figure 5 shows that relatively few participants used interdental brushes daily, while most reported never using them.

## 4. Discussion

### 4.1. Effectiveness of Individualized Education

Participation in the iTOP program led to significant short-term improvements in key clinical oral health parameters, especially plaque index and bleeding on probing. While improvements were observed at 3 months, by the 2-year follow-up, most periodontal parameters, except plaque index, had returned to baseline. The short-term gains were supported by statistical significance, effect sizes, and post hoc power, whereas long-term benefits were limited. These findings suggest that a single iTOP session, without ongoing reinforcement, may be insufficient to sustain long-term periodontal benefits.

This study was intentionally designed as a single-session intervention to assess the isolated effect of an initial iTOP training, reflecting pragmatic curricular constraints where repeated sessions may be difficult to implement. The decline in effect may be due to the lack of structured reinforcement following the initial session and the high academic workload and stress faced by medical and dental students, which could deprioritize daily oral hygiene. Another explanation is the general tendency for behavioral gains to dissipate without ongoing motivation or environmental support. Since both groups showed some improvements over time, especially in plaque index, the sustained effect in the iTOP group at 2 years may reflect not only the intervention but also academic progression, enhanced oral health literacy, and curricular exposure. Consequently, long-term differences should be interpreted cautiously.

In the broader context of oral hygiene education, various protocols—ranging from classroom-based group lectures to school-based reinforcement and digital app–based motivational programs—have consistently produced short-term improvements in plaque control and gingival health. For instance, Jönsson et al. [1] reported clinically meaningful plaque reductions after individually tailored education for periodontal patients, while school-based repeated sessions have been shown to maintain improved oral hygiene scores over short follow-ups [19]. A recent structured group-education trial demonstrated significant short-term improvements, which diminished without reinforcement. [2]. These results are consistent with the broader literature: iTOP’s individualized, hands-on approach produces notable initial effects, though sustained benefits likely require repeated reinforcement sessions. Long-term adherence to iTOP may be affected by individual and contextual factors such as changing intrinsic motivation, limited time, and competing academic demands. Previous studies have shown that knowledge alone does not guarantee sustained oral hygiene behavior among health profession students [5,6,12], and psychosocial stressors may further reduce compliance [13,14]. Understanding these barriers is essential for designing interventions that achieve lasting behavior change. Future studies should assess the scalability, cost-effectiveness, and optimal frequency of booster sessions needed to maintain iTOP’s benefits within university curricula.

The non-iTOP group showed initial improvements, but these effects were not sustained over time. The most pronounced deterioration was observed in bleeding on probing and probing depth. These findings suggest the added value of individualized training. Although other parameters worsened, the plaque index decrease in the control group likely reflects academic progression, improved oral health literacy, and curricular exposure, rather than the intervention itself. Academic progression may partly explain this finding, since earlier studies show a link between higher education and better oral health. Higher education levels are consistently linked with increased health literacy, improved oral hygiene practices, and more favorable periodontal health [20,21,22,23].

Few cohort studies have examined how oral health changes over time after educational interventions. In a study by Kalevski et al. [17], dental students underwent an initial oral health assessment before the educational intervention. Follow-up assessments measured clinical changes in oral health parameters. Although significant improvements were reported, oral hygiene levels among dental students remained suboptimal [17]. A similar pattern was observed at the Faculty of Medicine, University of Mostar. Students in the iTOP group had significantly better clinical outcomes during the second and third assessments than their peers in the control group. Nevertheless, there remains room for improvement in overall oral hygiene and clinical indicators. While previous studies have examined oral health education among dental students in Southeast Europe, to our knowledge, this appears to be among the first studies to assess the long-term clinical effectiveness of a single-session iTOP intervention in both medical and dental students.

A systematic review by Nakre et al. [18] found that short-term gains in oral health behaviors often diminish without sustained efforts, supporting the findings of this study. In the group that participated in the iTOP program, all measured parameters—except plaque index—returned to baseline values by the end of the 2-year follow-up. Although a statistically significant improvement in oral hygiene was observed at the three-month follow-up, this effect was no longer evident after two years. The findings indicate that regular education may be important for achieving lasting behavior change and better hygiene.

The findings align with health behavior theories, such as the Health Belief Model and the Theory of Planned Behavior [24,25], which highlight perceived benefits, self-efficacy, and ongoing external reinforcement as critical for maintaining long-term behavioral adherence. Integrating these theoretical insights can inform the development of more effective oral health education interventions aimed at maintaining improved hygiene behaviors over time.

### 4.2. Differences by Academic Program

At baseline, dental students had better periodontal indicators than medical students. Similar findings have been reported in previous studies, with dental students consistently showing superior oral hygiene knowledge, attitudes, and behaviors compared with their medical counterparts [26,27,28]. However, in this study, group differences were minimal, with significant variation only in probing depth and attachment level. By the 2-year follow-up, these differences had diminished, indicating a convergence in periodontal status between the student groups over time.

### 4.3. Impact of Academic Year

Baseline data showed that older students had significantly lower plaque index than younger students. This may reflect better oral hygiene habits among more advanced students. No significant differences were observed in other periodontal parameters between the two academic levels. After two years, when the cohorts reached their third and sixth years, the groups showed no significant differences in oral health.

Most previous studies have focused on comparing oral health knowledge between medical and dental students, analyzing differences in their understanding and attitudes. Within these groups, particular attention has been given to comparing the knowledge and attitudes of junior versus senior students. Studies in the general population suggest that younger individuals often have better oral health than older adults [29]. However, studies of medical and dental students have produced inconsistent findings regarding their knowledge and attitudes toward oral health. Yao et al. [30] found no significant differences in oral health knowledge and attitudes between first-year medical and dental students, although the latter had marginally higher scores. In addition, third-year dental students outperformed their medical peers in the same parameters, with statistically significant differences [30]. Other studies assessing medical students’ knowledge and attitudes have revealed a generally low level of awareness on this topic. Nevertheless, senior students showed significantly better awareness than junior ones. [31].

Additionally, studies analyzing dental students’ attitudes toward oral health and their personal oral hygiene practices have included both preclinical and clinical students. However, findings across these studies are inconsistent. Some report no significant differences between preclinical and clinical students [32], while others indicate that preclinical students have lower oral health knowledge, attitudes, and behaviors [33]. This highlights the need for structured educational interventions.

Such variations may be due to differences in target populations (e.g., medical vs. dental students), as well as disparities in conceptual frameworks, methodology, questionnaire types, and sample sizes.

### 4.4. Smoking Behavior Trends

Smoking prevalence increased during this study. Specifically, during the first assessment, 19.51% of participants reported being smokers, while by the third assessment—conducted two years later—this number had risen to 26.32%. This increase may reflect the high stress levels experienced by medical and dental students during academic training [34,35]. However, other studies show no statistical link between smoking and student age, suggesting that academic year may not reliably predict smoking behavior [36,37]. The lack of adequate smoking prevention and cessation programs at universities further worsens the situation. It is essential to develop prevention and education strategies aimed at reducing smoking rates among students [38]. Smoking has numerous negative effects not only on oral health but also on overall health [39]. This increase in smoking prevalence may have adversely influenced clinical outcomes, especially bleeding on probing and probing depth. Given that smoking is a well-known risk factor for periodontal disease, the increase in smoking prevalence may have partially attenuated the long-term effects of the iTOP intervention. Future studies should carefully account for smoking status as a potential confounder. While larger sample sizes may facilitate more comprehensive multivariate modeling, the influence of smoking on intervention effects should be interpreted with caution given the limitations of observational designs.

### 4.5. Study Limitations and Future Directions

The findings of this study are generally consistent with previous research, although no directly comparable study was identified. Adjusted mixed-model analyses yielded the same main conclusions as the non-parametric analyses, supporting the consistency of our findings. As with all research, several limitations should be acknowledged. Aligned with descriptive and model-based analyses, changes in probing depth and attachment level were small and not statistically significant, suggesting that the primary benefit of the intervention was in plaque control. First, this study was conducted at a single institution, which may limit the generalizability of the findings to other educational settings or cultural contexts. In addition, the study population consisted exclusively of medical and dental students, who may be more motivated and knowledgeable about oral hygiene than the general public. This may have led to greater adherence to oral hygiene recommendations and, consequently, to somewhat more favorable clinical outcomes than would be expected in broader, more heterogeneous populations. Furthermore, while medical and dental students represent a relevant and high-impact group, future studies should aim to replicate these findings in other academic or community populations to enhance the external validity and generalizability of the intervention. Second, the sample size was relatively small and constrained by the number of students available per academic year. Moreover, the sample included only first/fourth- and third/sixth-year students from both medicine and dentistry programs. Furthermore, academic progression and increased curricular exposure over the 2-year follow-up may have contributed to improvements in oral health parameters in both groups, potentially confounding the observed long-term difference in plaque index. Another limitation is the Hawthorne effect, whereby participants may have improved their behavior simply because they were being observed. Repeated professional cleanings in both groups may have further amplified this effect, potentially contributing to improvements independent of the intervention. Additionally, the single-site academic setting and culturally homogeneous sample limit the generalizability of our findings. Additionally, all participants received standardized oral hygiene kits at each assessment point, which may have inadvertently influenced the control group’s behavior and diminished observable differences between the intervention and control groups. This potential bias should be considered when interpreting the results. This study assessed the effects of a single-session iTOP with only a brief 3-month reinforcement; no ongoing reinforcement beyond that was provided. Long-term adherence to the techniques was not systematically monitored, and no objective compliance measures were obtained beyond baseline self-reports, limiting attribution of 2-year outcomes solely to the intervention. Therefore, the observed decline should be viewed in light of this limitation, and outcomes could potentially differ with repeated interventions.

Although we made efforts to standardize conditions across participants, we were unable to control for all potential confounding factors. These include individual motivation, differences in curricular exposure, use of additional hygiene tools, academic stress, personal health beliefs, and other lifestyle-related behaviors. Such unmeasured variables may have influenced the outcomes and should be carefully considered in future research designs. Furthermore, the local context in which this study was conducted may have influenced the results. Factors such as the local education system, the availability of preventive dental services, and cultural attitudes toward oral hygiene may significantly influence oral health behaviors and outcomes. These contextual factors limit the generalizability of our findings to other academic or geographical settings.

Although the final response rate was high (92.7%), a small number of non-responders may still introduce attrition bias.

Future studies of this type should aim to increase the sample size and ideally include all students at the Faculty of Medicine, University of Mostar. Multiple studies have confirmed that knowledge about oral health tends to improve with age and level of education [20,21,22,23]. To ensure this trend is reflected at the University of Mostar, ongoing education and awareness campaigns on oral health should be maintained.

Given the high prevalence of oral diseases such as caries, gingivitis, and periodontitis, targeted research in specific populations, e.g., schoolchildren and university students, is essential [40,41]. Such studies help assess oral health awareness and support the development of preventive and educational interventions. Although results may not be immediate, investing in education is a long-term strategy for improving oral health among future generations [42]. Educating young individuals on proper hygiene and the importance of oral health has the potential to positively influence not only their own long-term health, but also the health of those around them, particularly within their future families and professional roles.

## 5. Conclusions

This study suggests that Individually Trained Oral Prophylaxis (iTOP) is effective in achieving short-term improvements in oral health among medical and dental students. Plaque index was the only parameter showing a sustained effect at 2 years, which should be interpreted cautiously, as improvements in both groups may reflect academic progression and enhanced oral health literacy rather than the intervention alone. Other parameters, such as probing depth and bleeding on probing, reverted to baseline levels. These findings underscore the need for continuous reinforcement of oral health education. A single session appears insufficient for lasting behavioral change; therefore, future programs should include repeated or supplemental interventions. Differences between subgroups decreased over time, which may reflect both the intervention and academic progression. Overall, iTOP led to short-term improvements and a sustained effect on plaque index, supported by effect sizes and post hoc power, whereas other periodontal parameters reverted to baseline. iTOP appears promising as an educational intervention, though its incorporation into university curricula warrants cautious consideration. Due to the single-center design and limited sample size, these findings are preliminary, and further multicenter studies are warranted before wider implementation.

## Figures and Tables

**Table 1:** Differences in periodontal parameters between medical and dental students at all three time points.

		Field of Study						
	Dental Medicine			Medicine			Z	*p*
	M	IR	95% CI	M	IR	95% CI		
Average probing depth 1	1.80	0.3	1.7; 1.9	1.90	0.2	1.9; 1.95	−2.450	0.014
Average probing depth 2	1.75	0.2	1.7; 1.8	1.80	0.2	1.7; 1.85	−1.392	0.164
Average probing depth 3	1.90	0.1	1.9; 1.9	1.90	0.2	1.9; 2.0	−0.789	0.430
Average attachment level 1	−1.75	0.3	−1.9; −1.7	−1.90	0.2	−1.95; −1.9	2.491	0.013
Average attachment level 2	−1.75	0.2	−1.8; −1.7	−1.80	0.2	−1.85; −1.7	1.313	0.189
Average attachment level 3	−1.90	0.1	−1.9; −1.9	−1.90	0.2	−2.0; −1.9	0.868	0.385
Plaque 1	69.00	22	63.0; 76.0	69.00	29	64.0; 73.0	0.023	0.981
Plaque 2	31.50	26	20.0; 34.0	31.50	33	26.0; 39.0	−0.515	0.606
Plaque 3	18	25	13.0; 32.0	23.50	19	16.5; 28.5	−0.531	0.596
Bleeding on probing 1	6.50	8	4.0; 9.0	7.00	7	5.0; 9.5	−1.382	0.167
Bleeding on probing 2	4.00	5	3.0; 5.0	4.00	6	3.5; 7.0	−0.804	0.422
Bleeding on probing 3	8.50	9	7.0; 12.0	10.00	8	8.0; 11.0	−0.339	0.735

Time points are labeled as: (1) baseline, (2) 3-month follow-up, and (3) 2-year follow-up.

**Table 2:** Differences between younger and older students in periodontal parameters at all three time points.

		Year of Study						
	1st/4th			3rd/6th			Z	*p*
	M	IR	95% CI	M	IR	95% CI		
Average probing depth 1	1.80	0.3	1.8; 1.95	1.90	0.3	1.8; 1.9	0.062	0.951
Average probing depth 2	1.80	0.3	1.7; 1.85	1.80	0.1	1.7; 1.8	0.644	0.520
Average probing depth 3	1.90	0.1	1.9; 2.0	1.90	0.2	1.9; 2.0	0.591	0.554
Average attachment level 1	−1.80	0.3	−1.9; −1.75	−1.90	0.3	−1.9; −1.8	0.370	0.711
Average attachment level 2	−1.80	0.3	−1.85; −1.7	−1.80	0.1	−1.8; −1.7	−0.403	0.687
Average attachment level 3	−1.90	0.1	−2.0; −1.9	−1.90	0.2	−2.0; −1.9	−0.475	0.635
Plaque 1	74.50	27	66.0; 83.5	67.00	22	62.0; 72.0	2.093	0.036
Plaque 2	32.50	23	27.5; 37.0	27.00	37	18.5; 35.0	0.924	0.356
Plaque 3	23.00	31	13.5; 32.5	18.50	20	14.5; 26.0	0.740	0.460
Bleeding on probing 1	8.00	9	5.0; 11.0	5.00	7	4.5; 8.0	1.298	0.194
Bleeding on probing 2	4.00	7	4.0; 7.0	4.00	6	3.0; 6.0	0.888	0.375
Bleeding on probing 3	10	10	7.0; 14.0	9	6	7.5; 11.0	0.444	0.657

Time points are labeled as: (1) baseline, (2) 3-month follow-up, and (3) 2-year follow-up.

**Table 3:** Intra-group comparisons of periodontal parameters in iTOP and non-iTOP groups at baseline, 3-month, and 2-year follow-ups.

		Measurement Results					Difference		Effect Size
Group	$\bar{X}$	SD	95% CI	Quartiles			F	*p*	η^2^p
				25	50	75			
iTOP									
Average probing depth 1	1.85	0.21	1.78; 1.92	1.70	1.90	2.00			
Average probing depth 2	1.74	0.17	1.69; 1.80	1.60	1.70	1.80	16.27	<0.001	0.274
Average probing depth 3	1.92	0.15	1.87; 1.97	1.80	1.90	2.00			
Average attachment level 1	−1.85	0.20	−1.92; −1.79	−2.00	−1.90	−1.70			
Average attachment level 2	−1.74	0.17	−1.79; −1.69	−1.80	−1.70	−1.60	14.26	<0.001	0.028
Average attachment level 3	−1.81	0.63	−2.02; −1.60	−2.00	−1.90	−1.80			
Plaque 1	67.60	19.21	61.46; 73.74	61.00	67.00	83.50			
Plaque 2	23.35	13.18	19.14; 27.56	13.00	19.00	33.00	65.82	<0.001	0.748
Plaque 3	17.46	14.52	12.62; 22.30	10.00	13.00	18.00			
Bleeding on probing 1	8.72	6.49	6.65; 10.80	4.25	8.50	11.00			
Bleeding on probing 2	4.53	3.94	3.27; 5.78	2.00	3.00	4.00	18.04	<0.001	0.230
Bleeding on probing 3	7.41	3.25	6.32; 8.49	5.00	7.00	9.25			
Non-iTOP									
Average probing depth 1	1.88	0.17	1.82; 1.93	1.70	1.90	2.00			
Average probing depth 2	1.78	0.14	1.74; 1.82	1.70	1.80	1.90	20.95	<0.001	0.344
Average probing depth 3	1.95	0.16	1.90; 2.00	1.90	1.90	2.00			
Average attachment level 1	−1.88	0.16	−1.93; −1.83	−2.00	−1.90	−1.70			
Average attachment level 2	−1.78	0.14	−1.82; −1.74	−1.90	−1.80	−1.70	21.84	<0.001	0.358
Average attachment level 3	−1.97	0.15	−2.01; −1.91	−2.00	−1.90	−1.90			
Plaque 1	67.38	20.10	61.12; 73.65	56.00	71.50	82.75			
Plaque 2	44.55	23.32	37.28; 51.81	26.00	39.50	57.25	25.31	<0.001	0.465
Plaque 3	35.08	18.66	29.03; 41.12	22.25	32.00	41.75			
Bleeding on probing 1	8.14	6.89	5.99; 10.29	3.75	5.50	10.25			
Bleeding on probing 2	7.90	5.97	6.04; 9.77	4.00	6.50	10.00	16.16	<0.001	0.247
Bleeding on probing 3	13.59	5.90	11.68; 15.50	9.25	14.00	16.75			

Time points are labeled as: (1) baseline, (2) 3-month follow-up, and (3) 2-year follow-up.

**Table 4:** Comparison of clinical parameters between the iTOP and non-iTOP groups at each time point.

		Group							Effect Size	
	iTOP			Non-iTOP			Z	*p*	Cliff’s δ	|r|
	M	IR	95% CI	M	IR	95% CI				
Average probing depth 1	1.900	0.3	1.75; 1.9	1.900	0.3	1.8; 1.9	−0.563	0.574	−0.071	0.061
Average probing depth 2	1.700	0.2	1.7; 1.8	1.800	0.2	1.7; 1.8	−1.189	0.235	−0.149	0.129
Average probing depth 3	1.900	0.2	1.9; 1.9	1.900	0.1	1.9; 2.0	−1.278	0.201	−0.165	0.142
Average attachment level 1	−1.900	0.3	−1.9; −1.7	−1.900	0.3	−1.9; −1.8	0.693	0.489	0.087	0.075
Average attachment level 2	−1.700	0.2	−1.8; −1.7	−1.800	0.2	−1.8; −1.7	1.408	0.159	0.177	0.152
Average attachment level 3	−1.900	0.2	−1.9; −1.9	−1.900	0.1	−2.0; −1.9	1.651	0.099	0.212	0.182
Plaque 1	67.00	22	65.0; 74.5	71.50	26	61.5; 76.0	0.070	0.945	0.009	0.008
Plaque 2	19.00	20	14.5; 31.0	39.50	29	32.5; 52.0	−4.418	<0.001	−0.567	0.488
Plaque 3	13.00	8	10.0; 14.0	32.00	20	26.0; 39.0	−5.068	<0.001	−0.675	0.581
Bleeding on probing 1	8.50	7	5.0; 10.5	5.50	6	4.0; 8.0	0.814	0.416	0.104	0.090
Bleeding on probing 2	3.00	2	3.0; 4.0	6.50	6	4.5; 8.5	−3.234	0.001	−0.412	0.354
Bleeding on probing 3	7.00	5	6.0; 8.0	14.00	8	11.0; 15.0	−5.087	<0.001	−0.676	0.582

Time points are labeled as: (1) baseline, (2) 3-month follow-up, and (3) 2-year follow-up.

**Table 5:** Adjusted mixed-model results.

Outcome	Time	Adj Mean iTOP (95% CI)	Adj Mean Control (95% CI)	Difference (iTOP–Control) (95% CI)	*p*-Value
APD (mm)	T2	1.76 (1.70–1.83)	1.79 (1.73–1.86)	−0.03 (−0.10–0.05)	0.456
APD (mm)	T3	1.94 (1.88–2.01)	1.97 (1.90–2.04)	−0.02 (−0.10–0.05)	0.538
AAL (mm)	T2	−1.76 (−1.87–−1.65)	−1.80 (−1.90–−1.69)	0.03 (−0.09–0.16)	0.594
AAL (mm)	T3	−1.84 (−1.95–−1.72)	−1.97 (−2.08–−1.87)	0.14 (0.00–0.27)	0.043
Plaque (%)	T2	17.67 (12.52–23.88)	39.93 (30.80–49.90)	−22.26 (−32.15–−12.90)	<0.001
Plaque (%)	T3	13.23 (9.05–18.57)	28.63 (21.03–37.35)	−15.40 (−24.21–−7.41)	<0.001
BOP (%)	T2	4.17 (3.15–5.37)	6.86 (5.26–8.81)	−2.70 (−4.57–−1.05)	0.001
BOP (%)	T3	7.90 (6.04–10.11)	13.55 (10.55–17.10)	−5.65 (−9.13–−2.50)	<0.001

Primary analysis from linear mixed-effects models adjusted for program, academic year, smoking, and baseline oral-hygiene habits. Estimates for plaque and BOP are on the percentage point (pp) scale; APD and AAL are in millimeters.

**Table 6:** Results of Omnibus Group × Time Interaction Tests for Key Clinical Outcomes.

Outcome	F-Stat (df)	*p*-Value
APD (mm)	F(2, 240) = 0.78	0.460
AAL (mm)	F(2, 240) = 3.52	0.031
Plaque (%)	F(2, 240) = 22.41	<0.001
BOP (%)	F(2, 240) = 7.88	<0.001

## Data Availability

The data that support the findings of this study are available upon request from the authors.

## Abbreviations

The following abbreviations are used in this manuscript:

iTOP	Individually trained oral prophylaxis
APD	Average probing depth
AAL	Average attachment level
BOP	Bleeding on probing
PLQ	Plaque index
CI	Confidence interval
IQR	Interquartile range
CEJ	Cementoenamel junction
UNC-15	University of North Carolina 15 probe
pp	percentage points

## Appendix A

Post hoc power analyses were conducted for the main between-group comparisons (iTOP vs. control, dental vs. medical students, and younger vs. older students) for each primary outcome across time points. The analyses report the observed effect size (Cohen’s *d*) and the corresponding post hoc statistical power (1 − β), calculated for the actual sample sizes using a two-tailed independent-samples *t*-test with α = 0.05. In this context, 1 − β represents the probability of detecting the observed effect given the sample size; values ≥ 0.80 are generally considered to indicate adequate statistical power.

**Table A1:** Post hoc power (1 − β) and Cohen’s *d* for between-group comparisons: iTOP vs. control group.

Comparison	Timepoint	Outcome	Group 1	Group 2	Group 1 Mean CI	Group 2 Mean CI	Cohen’s *d*	Post Hoc Power Test
iTOP group versus control group	T1	APD	40	42	1.85 (95% CI: 1.78, 1.92)	1.88 (95% CI: 1.82, 1.93)	−0.14	0.10
	T1	AAL	40	42	−1.85 (95% CI: −1.92, −1.79)	−1.88 (95% CI: −1.93, −1.83)	0.13	0.09
	T1	PLQ	40	42	67.60 (95% CI: 61.46, 73.74)	67.38 (95% CI: 61.12, 73.65)	0.01	0.05
	T1	BOP	40	42	8.72 (95% CI: 6.65, 10.80)	8.14 (95% CI: 5.99, 10.29)	0.09	0.07
	T2	APD	40	42	1.74 (95% CI: 1.69, 1.80)	1.78 (95% CI: 1.74, 1.82)	−0.23	0.18
	T2	AAL	40	42	−1.74 (95% CI: −1.79, −1.69)	−1.78 (95% CI: −1.82, −1.74)	0.25	0.20
	T2	PLQ	40	42	23.35 (95% CI: 19.14, 27.56)	44.55 (95% CI: 37.28, 51.81)	−1.11	1.00
	T2	BOP	40	42	4.53 (95% CI: 3.27, 5.78)	7.90 (95% CI: 6.04, 9.77)	−0.66	0.84
	T3	APD	37	39	1.92 (95% CI: 1.87, 1.97)	1.95 (95% CI: 1.90, 2.00)	−0.23	0.16
	T3	AAL	37	39	−1.81 (95% CI: −2.02, −1.60)	−1.96 (95% CI: −2.01, −1.91)	0.32	0.28
	T3	PLQ	37	39	17.46 (95% CI: 12.62, 22.30)	35.08 (95% CI: 29.03, 41.12)	−1.05	0.99
	T3	BOP	37	39	7.41 (95% CI: 6.32, 8.49)	13.59 (95% CI: 11.68, 15.50)	−1.29	1.00

Time points are labeled as: (T1) baseline, (T2) 3-month follow-up, and (T3) 2-year follow-up. Outcomes are labeled as: (APD) average probing depth, (AAL) average attachment level, (PLQ) plaque, and (BOP) bleeding on probing.

**Table A2:** Post hoc power (1 − β) and Cohen’s *d* for between-group comparisons: dental students vs. medical students.

Comparison	Timepoint	Outcome	Group 1	Group 2	Group 1 Mean CI	Group 2 Mean CI	Cohen’s *d*	Post Hoc Power Test
Dental students versus Medical students	T1	APD	40	42	1.82 (95% CI: 1.75, 1.88)	1.91 (95% CI: 1.86, 1.96)	−0.49	0.59
	T1	AAL	40	42	−1.82 (95% CI: −1.89, −1.76)	−1.90 (95% CI: −1.95, −1.86)	0.47	0.55
	T1	PLQ	40	42	67.42 (95% CI: 60.91, 73.94)	67.55 (95% CI: 61.63, 73.47)	−0.01	0.05
	T1	BOP	40	42	7.22 (95% CI: 5.50, 8.95)	9.57 (95% CI: 7.21, 11.93)	−0.36	0.36
	T2	APD	40	42	1.74 (95% CI: 1.69, 1.78)	1.78 (95% CI: 1.73, 1.83)	−0.30	0.27
	T2	AAL	40	42	−1.74 (95% CI: −1.79, −1.69)	−1.78 (95% CI: −1.83, −1.73)	0.29	0.25
	T2	PLQ	40	42	32.62 (95% CI: 26.02, 39.23)	35.71 (95% CI: 28.59, 42.84)	−0.14	0.10
	T2	BOP	40	42	5.47 (95% CI: 4.07, 6.88)	7.00 (95% CI: 5.11, 8.89)	−0.29	0.25
	T3	APD	38	38	1.92 (95% CI: 1.87, 1.97)	1.95 (95% CI: 1.90, 2.00)	−0.20	0.14
	T3	AAL	38	38	−1.92 (95% CI: −1.96, −1.87)	−1.86 (95% CI: −2.06, −1.65)	−0.14	0.09
	T3	PLQ	38	38	27.13 (95% CI: 20.06, 34.20)	25.87 (95% CI: 20.58, 31.15)	0.07	0.06
	T3	BOP	38	38	10.18 (95% CI: 8.51, 11.86)	10.97 (95% CI: 8.91, 13.04)	−0.14	0.09

Time points are labeled as: (T1) baseline, (T2) 3-month follow-up, and (T3) 2-year follow-up. Outcomes are labeled as: (APD) average probing depth, (AAL) average attachment level, (PLQ) plaque, and (BOP) bleeding on probing.

**Table A3:** Post hoc power (1 − β) and Cohen’s *d* for between-group comparisons: younger students vs. older students.

Comparison	Timepoint	Outcome	Group 1	Group 2	Group 1 Mean CI	Group 2 Mean CI	Cohen’s *d*	Post Hoc Power Test
Younger students versus older students	T1	APD	40	42	1.86 (95% CI: 1.80, 1.91)	1.87 (95% CI: 1.81, 1.93)	−0.06	0.06
	T1	AAL	40	42	−1.85 (95% CI: −1.90, −1.80)	−1.88 (95% CI: −1.94, −1.82)	0.16	0.11
	T1	PLQ	40	42	72.05 (95% CI: 65.73, 78.37)	63.14 (95% CI: 57.36, 68.93)	0.47	0.55
	T1	BOP	40	42	9.88 (95% CI: 7.29, 12.46)	7.05 (95% CI: 5.60, 8.50)	0.43	0.49
	T2	APD	40	42	1.77 (95% CI: 1.72, 1.83)	1.75 (95% CI: 1.71, 1.79)	0.15	0.10
	T2	AAL	40	42	−1.76 (95% CI: −1.82, −1.71)	−1.75 (95% CI: −1.80, −1.71)	−0.07	0.06
	T2	PLQ	40	42	36.58 (95% CI: 29.27, 43.88)	31.95 (95% CI: 25.52, 38.39)	0.21	0.16
	T2	BOP	40	42	6.97 (95% CI: 5.01, 8.94)	5.57 (95% CI: 4.20, 6.94)	0.26	0.22
	T3	APD	36	40	1.95 (95% CI: 1.89, 2.00)	1.92 (95% CI: 1.87, 1.97)	0.16	0.10
	T3	AAL	36	40	−1.84 (95% CI: −2.06, −1.63)	−1.93 (95% CI: −1.97, −1.88)	0.18	0.12
	T3	PLQ	36	40	30.00 (95% CI: 22.24, 37.76)	23.35 (95% CI: 18.92, 27.78)	0.36	0.33
	T3	BOP	36	40	11.36 (95% CI: 9.02, 13.70)	9.88 (95% CI: 8.50, 11.25)	0.26	0.20

Time points are labeled as: (T1) baseline, (T2) 3-month follow-up, and (T3) 2-year follow-up. Outcomes are labeled as: (APD) average probing depth, (AAL) average attachment level, (PLQ) plaque, and (BOP) bleeding on probing.
